# Nano‐TiO_2_ penetration of oral mucosa: *in vitro* analysis using 3D organotypic human buccal mucosa models

**DOI:** 10.1111/jop.12469

**Published:** 2016-07-08

**Authors:** Victoria Konstantinova, Mohamed Ibrahim, Stein A. Lie, Eivind Salmorin Birkeland, Evelyn Neppelberg, Mihaela Cuida Marthinussen, Daniela Elena Costea, Mihaela R. Cimpan

**Affiliations:** ^1^ Department of Clinical DentistryFaculty of Medicine and DentistryUniversity of BergenBergenNorway; ^2^ Gade Laboratory for PathologyDepartment of Clinical MedicineFaculty of Medicine and DentistryUniversity of BergenBergenNorway; ^3^ Department of Global Public Health and Primary CareCentre for International HealthFaculty of Medicine and DentistryUniversity of BergenBergenNorway; ^4^ Department of Oral SurgeryInstitute of Clinical DentistryUniversity of BergenBergenNorway; ^5^ Department of Ear‐Nose‐and‐Throat SurgeryHaukeland University HospitalBergenNorway; ^6^ Oral Health Centre of Expertise in Western NorwayHordalandNorway; ^7^ Department of PathologyHaukeland University HospitalBergenNorway

**Keywords:** epithelium, nanoparticles, oral, organotypic model, titanium dioxide

## Abstract

**Background:**

Oral cavity is a doorway for a variety of products containing titanium dioxide (TiO_2_) nanoparticles (NPs) (nano‐TiO_2_) such as food additives, oral healthcare products and dental materials. Their potential to penetrate and affect normal human oral mucosa is not yet determined.

**Objectives:**

To evaluate the ability of nano‐TiO_2_ to penetrate the *in vitro* reconstructed normal human buccal mucosa (RNHBM).

**Methods:**

RNHBM was generated from primary normal human oral keratinocytes and fibroblasts isolated from buccal oral mucosa of healthy patients (*n* = 6). The reconstructed tissues were exposed after 10 days to clinically relevant concentrations of spherical or spindle rutile nano‐TiO_2_ in suspension for short (20 min) and longer time (24 h). Ultrahigh‐resolution imaging (URI) microscopy (CytoViva^™^, Auburn, AL, USA) was used to assess the depth of penetration into reconstructed tissues.

**Results:**

Ultrahigh‐resolution imaging microscopy demonstrated the presence of nano‐TiO_2_ mostly in the epithelium of RNHBM at both 20 min and 24‐h exposure, and this was shape and doze dependent at 24 h of exposure. The depth of penetration diminished in time at higher concentrations. The exposed epithelium showed increased desquamation but preserved thickness.

**Conclusion:**

Nano‐TiO_2_ is able to penetrate RNHBM and to activate its barrier function in a doze‐ and time‐dependent manner.

## Introduction

The unusual properties of nanomaterials (NMs) led to their use in, among others, oral hygiene products, food additives and dental materials 1, 2, 3. Their increased use raises concerns regarding the impact on biological systems 1, 2, 4.

Titanium dioxide (TiO_2_) is one of the most used materials 5, due to its desirable properties. In bulk form, TiO_2_ exhibits corrosion resistance, high biocompatibility, specific strength, low density, making it suitable for biomedical applications such as implants and vascular stents. At nano‐level, the specific high surface area and the quantum effects lead to an increased chemical reactivity 1, 2. Titanium dioxide occurs mainly in three crystalline forms: anatase, rutile and brookite, each of them being associated with different properties and toxicological effects 5, 6. Increased use of nano‐TiO_2_ in paints, cosmetics, dental care products and food additives elevates the exposure risk via ingestion, inhalation and possible penetration through the oral mucosal tissues as well as skin. Oral mucosa is exposed to an array of NMs from various sources, oral intake of TiO_2_ particles of 100–200 nm being reported to be considerably high 3. In UK, dietary consumption of TiO_2_ has been estimated to be approx. 5 mg/person/day 3, 7. Although considered less hazardous than other NMs, nano‐TiO_2_ is non‐degradable and can accumulate in different organs leading to chronic toxicity 2.

Being several folds more permeable than skin, oral mucosa is an attractive site for drug delivery and toxicity studies 8. There are three types of oral mucosa: lining, masticatory and specialized tongue mucosa 9. Lining mucosa covers the mobile structures, is not keratinized, more elastic, deformable and more permeable as opposed to masticatory mucosa that lines gingivae and hard palate. In addition to its loosely packed nature, this makes lining mucosa more susceptible to penetration by external objects 10. Lining (buccal) mucosa has been subject to a considerable amount of research, mostly from a drug delivery/pharmaceutical point of view, but the penetration potential of nano‐TiO_2_ and possible related toxicological risks have been very little addressed. The aim of this study was to assess the penetration of nano‐TiO_2_ into human buccal mucosa by exposing *in vitro* 3D organotypic (OT) buccal mucosa models to two types of 40 nm rutile nano‐TiO_2_. The role of NM shape, that is spindle vs. spherical, was also evaluated.

## Materials and methods

### Nanoparticle characterization and preparation

**Figure 1:**
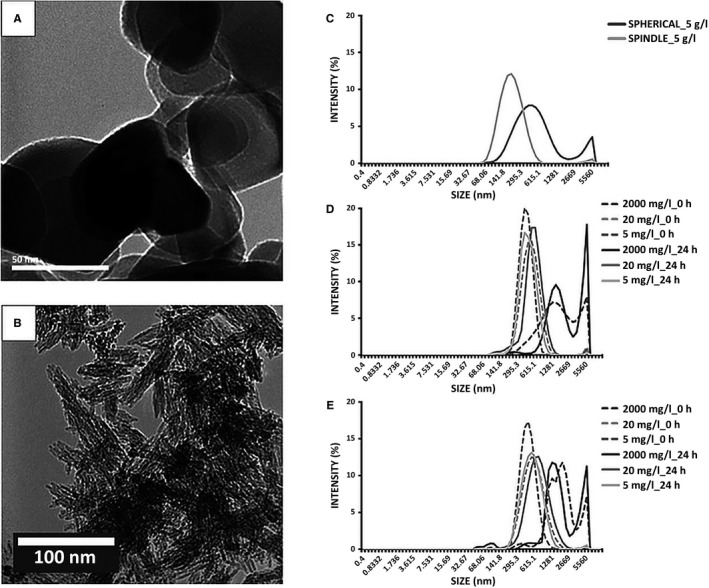
TEM images of the nano‐TiO_2_ powders: spherical nano‐TiO_2_ (A) and spindle nano‐TiO_2_ (B). Hydrodynamic size distribution as shown by the DLS measurements: batch suspension in distilled water for both types of particles (C), spherical nano‐TiO_2_ in FAD‐OT medium exposure media (time 0 and 24 h) (D) and spindle‐shaped nano‐TiO_2_ in FAD‐OT medium exposure media (time 0 and 24 h) (E).

Nano‐TiO_2_ is produced in a variety of shapes and sizes that are known to influence its interaction with cells 11. To study the role of NP shape, two types of rutile TiO_2_ NMs were used in this study: spherical, 40 nm, (American Elements^®^, Los Angeles, CA, USA) and spindle‐shaped, 40 × 10 nm, (Nanostructured & Amorphous Materials Inc., Garland, TX, USA) 6, (Fig. 1). The particle concentrations were 5, 20 and 2000 mg/l, which are relevant for oral exposure to TiO_2_ from various sources 7. Stock suspensions of 5 g/l nano‐TiO_2_ in deionized water were sonicated for 1 min with a 130‐Watt ultrasonic processor at 70% duty (VCX130, Vibra‐Cell, 130 W; Sonics & Materials Inc., Newtown, CT, USA) 12. Working suspensions were made by adding necessary volumes of culture medium immediately after sonication and rotated for at least 2 min. The NPs’ shape and size in powder were previously characterized 6. The hydrodynamic diameter (HD), polydispersity index (PDI) and ζ‐potential were measured using a Zetasizer Nano ZSP device (Malvern Instruments, Malvern, UK) both in stock solution and OT culture medium, the latter at time 0 and after 24 h.

### Isolation of primary cells and generation of reconstructed normal human buccal mucosa (RNHBM)

Samples of human normal buccal mucosa were obtained during wisdom tooth extraction after informed consent (*n* = 6 healthy volunteers) and used for isolation of normal oral fibroblasts and keratinocytes. The biopsy was cleaned and washed twice with Dulbecco's modified Eagle's medium (DMEM) (Sigma, St Louis, MO, USA) supplemented with 2% antibiotics–antimycotics (Gibco, Grand Island, NY, USA) for 5 min. Tissue was then kept for 24 h in 20 mg dispase in 7.5 ml DMEM with 2% antibiotics–antimycotics at +4°C. The following day epithelium was easily and completely separated from connective tissue with the help of tweezers. Fibroblasts were isolated from connective tissue explants and cultured in DMEM with 10% foetal bovine serum (FBS) and 1% antibiotics‐antimycotics. Keratinocytes were obtained by trypsinization of epithelium (10× Trypsin‐EDTA) (Sigma) and cultured in keratinocyte serum‐free medium (KSFM) supplemented with 1 ng/ml human recombinant epidermal growth factor, 25 μg/ml bovine pituitary extract (Gibco) and 1% antibiotics‐antimycotics. Both cell types were propagated and expanded in their specific culture medium for 2–4 passages 13. From each biopsy, a set of organotypic tissues was constructed from keratinocytes and fibroblasts from the same donor.

### Exposure of RNHBM to nano‐TiO_2_, tissue harvesting and processing

In order to avoid the leakage and to prevent exposing of organotypic tissue from the bottom (connective tissue part), a drop of only 20 μl of nano‐TiO_2_ suspended in medium was placed on the top of the model, precisely in the middle. Tissues in which the suspension poured over *t* edges were excluded from the analysis. Tissues were harvested within 20 min and after 24 h of exposure, washed in phosphate‐buffered saline (PBS) (Gibco) thrice and fixed with 10% buffered formalin for 24–48 h. RNHBM tissues were processed separately for each type of NPs and exposure time. Tissues were washed once in PBS, twice in distilled water, dehydrated in graded ethanol baths and then kept 30 min in xylene, followed by paraffin baths (Paraplast X‐TRA^®^). Histological sections (3 μm thickness) were cut with a sliding microtome (Leica SM2000R, Leica Biosystems Nussloch GmbH, Heidelberg, Germany), placed on glass slides cleaned with ethanol and then stained with Haematoxylin–Eosin in a Tissue‐Tek^®^ Prisma^™^ slide steiner (Sakura Seiki Co., Ltd, Nagano, Japan).

### Ultrahigh‐resolution microscopy and image analysis

Ultrahigh‐resolution imaging (URI) (CytoViva^™^, Auburn, AL, USA) was employed to assess penetration of nano‐TiO_2_ into RNHBM using a 100× oil immersion objective. CytoViva^™^ is a high contrast optical dark‐field system with an annular condenser; light is collimated without loss of intensity at oblique angles giving improved contrast and signal‐to‐noise ratio (90 nm resolution) 14. Nanoparticles and other light scattering objects appear as bright features on a dark background 15; therefore, the fluorescent marking of NPs, which might modify their physicochemical properties, is not needed. The average tissue length was approximately 10 mm. To reduce bias and obtain representative information from the tissues, when taking pictures, the peripheral parts (0.2 mm) of tissues were excluded and images were taken at a 1‐mm interval. A qualitative screening of the reconstructed mucosa of the OTs was performed before starting the measurements and the tissues without epithelium, with discontinuous epithelium, or with an epithelium less than 20 μm thickness, were excluded from the analysis. Image analysis was performed with NIS‐Elements software (v. 2.3, Nikon, Japan).

### Statistical analysis

Data were expressed as mean ± standard error of the mean (SEM). A general linear model was used to compare means within and between different treatment groups. A *P*‐value of <0.05 was considered statistically significant.

### Ethical considerations

A written informed consent was retrieved from each patient prior to the start of the study. The study was approved by Regional Committee for Medical and Health Research for Western Norway (2013/1492/REK Vest).

## Results

### Spindle‐shaped nano‐TiO_2_ formed smaller agglomerates and were more stable in suspension

The shape of nano‐TiO_2_ particles was confirmed by TEM (Fig. 1A,B, Table 1) as previously reported 6. Spherical nano‐TiO_2_ formed larger agglomerates in ^dd^H_2_O (459.93 ± 18.36 nm) compared to spindle‐shaped agglomerates (180.67 ± 2.25) (Fig. 1C, Table 1). In the medium, spherical particles also formed larger agglomerates, except at 2000 mg/l. Agglomerate sizes increased with time, larger agglomerates being registered after 24 h (Fig. 1D,E, Table 1). The ζ‐potential in ^dd^H_2_O revealed a higher absolute value for the spindle‐shaped particles, indicating their higher stability in suspension, a result that correlates well with their smaller agglomerates’ sizes. Nevertheless, the ζ‐potentials were relatively similar at all concentrations and over time in culture medium (Table 1).

**Table 1:** Physicochemical characteristics of nano‐TiO_2_ in powder form, water and culture medium

			Spherical Nano‐TiO2 (Ti2)			Spindle Nano‐TiO2 (Ti4)		
Supplier's description			Rutile 40 nm (TI‐OX‐02‐NP.050; American Elements^®^, USA)			Rutile 40 × 10 nm (#5480MR; Nanostructured & Amorphous Materials Inc., USA)		
S_BET_ (m^2^/g)^a^			38			165		
D_BET_ (nm)^b^			37			Not spherical		
Crystal structure			92% Rutile 8% Anatase			100% Rutile		
Crystal size (nm)^c^			Rutile: 21			Rutile: 8.5		
IEP^d^			–			3.10		
Circularity ± SD^e^			0.79 ± 0.08			0.29 ± 0.04		
MECD ± SD^f^			36 ± 22			14 ± 6		

			H.d.^g^	Pdi^h^	zeta^i^	H.d.	Pdi	zeta
Batch (in MiliQ water)	5 g/l		459.93 ± 18.36	0.41 ± 0.04	0.30 ± 1.10	180.67 ± 2.25	0.21 ± 0.01	−26.52 ± 0.80
In FAD‐OT medium	5 μg/ml	0 h	469.12 ± 30.62	0.45 ± 0.05	−8.55 ± 0.57	401.54 ± 9.50	0.38 ± 0.06	−9.70 ± 0.89
		24 h	545.01 ± 62.63	0.53 ± 0.04	−9.03 ± 0.65	404.65 ± 13.31	0.35 ± 0.04	−9.44 ± 0.80
	20 μg/ml	0 h	514.94 ± 24.31	0.31 ± 0.05	−8.83 ± 1.30	468.99 ± 7.23	0.26 ± 0.02	−9.45 ± 1.09
		24 h	671.58 ± 65.56	0.53 ± 0.07	−10.8 ± 0.8	571.10 ± 9.11	0.28 ± 0.03	−9.75 ± 0.55
	2000 μg/ml	0 h	1105.47 ± 183.29	0.38 ± 0.04	−9.11 ± 2.86	2389.35 ± 503.66	0.63 ± 0.21	−12.04 ± 0.99
		24 h	2079.12 ± 393.21	0.43 ± 0.08	−10.65 ± 1.54	3398.20 ± 460.93	0.51 ± 0.12	−12.60 ± 0.79

a Specific surface area.

b Calculated particle's diameter from BET measurements, DBET = *k*/*q*_SBET, *k* = 6 for a sphere. *q* is the density of the powder.

c Calculated using Scherrer equation: D = *kk*/*b*_cosh. A. (anatase, 101), R. (rutile, 110).

d Isoelectric point from titration curve: zeta potential vs. pH in aqueous solution of 0.14 M NaCl.

e Circularity from TEM pictures of NPs (from 0 to 1, where 1 is a perfect circle).

f Average microscopy equivalent circle diameter (nm) from TEM pictures of NPs.

g Hydrodynamic diameter.

h Polydispersion index.

i Zeta potential.

The physicochemical characteristics in powder form were reproduced form Allouni et al. 6, with permission.

### Spindle‐shaped nano‐TiO_2_ was found more superficially than spherical ones in the epithelium of RNHBM

**Figure 2:**
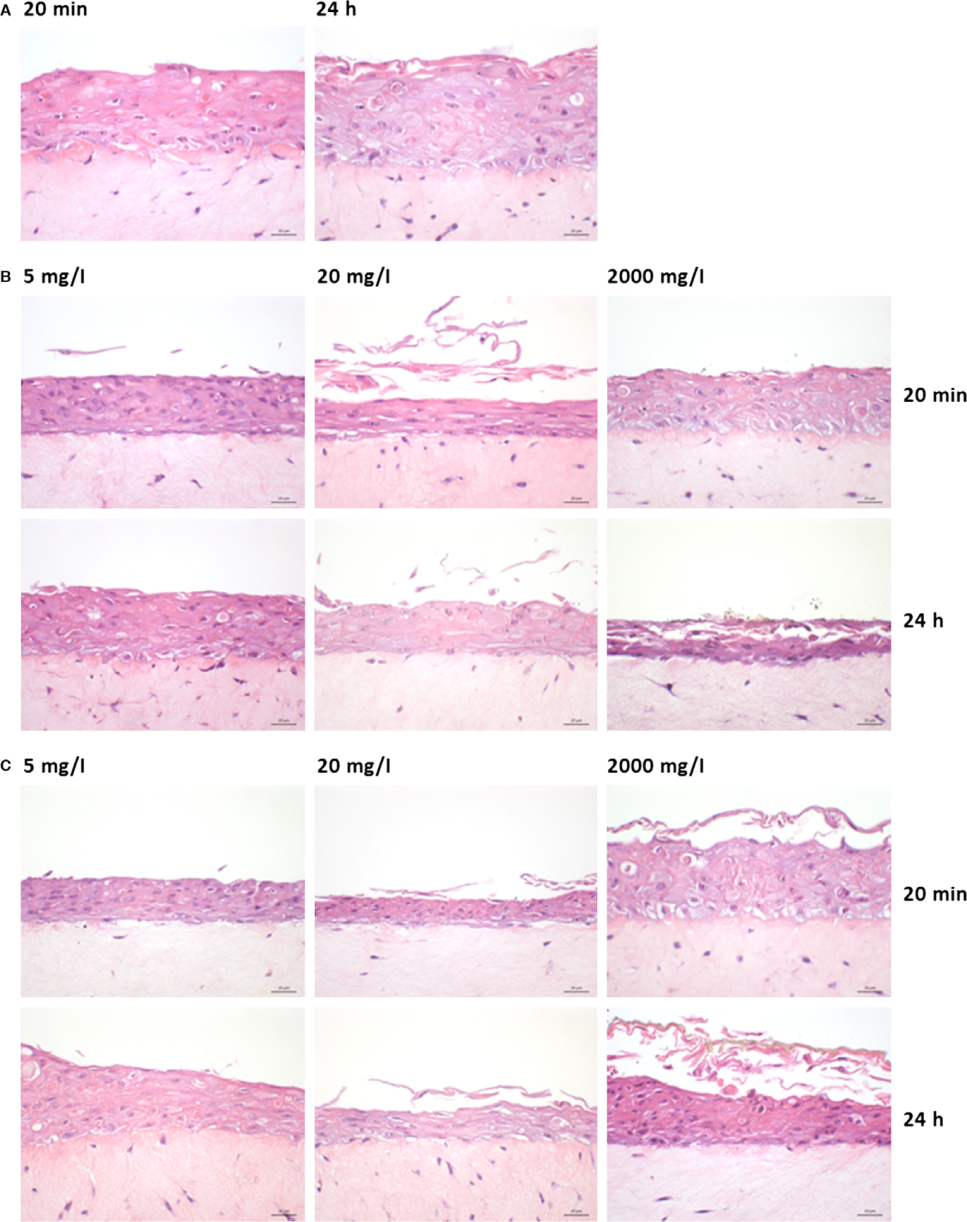
Representative light microscopy images of unexposed (A) and nano‐TiO_2_ exposed (B, C) 3D organotypic tissues for 20 min and 24 h. The reconstructed normal human oral buccal tissues were harvested, fixed in formalin, embedded in paraffin, sectioned and haematoxylin–eosin stained. Control (A), spherical nano‐TiO_2_ (B), spindle nano‐TiO_2_ (C). Scale‐bar: 20 μm.

**Figure 3:**
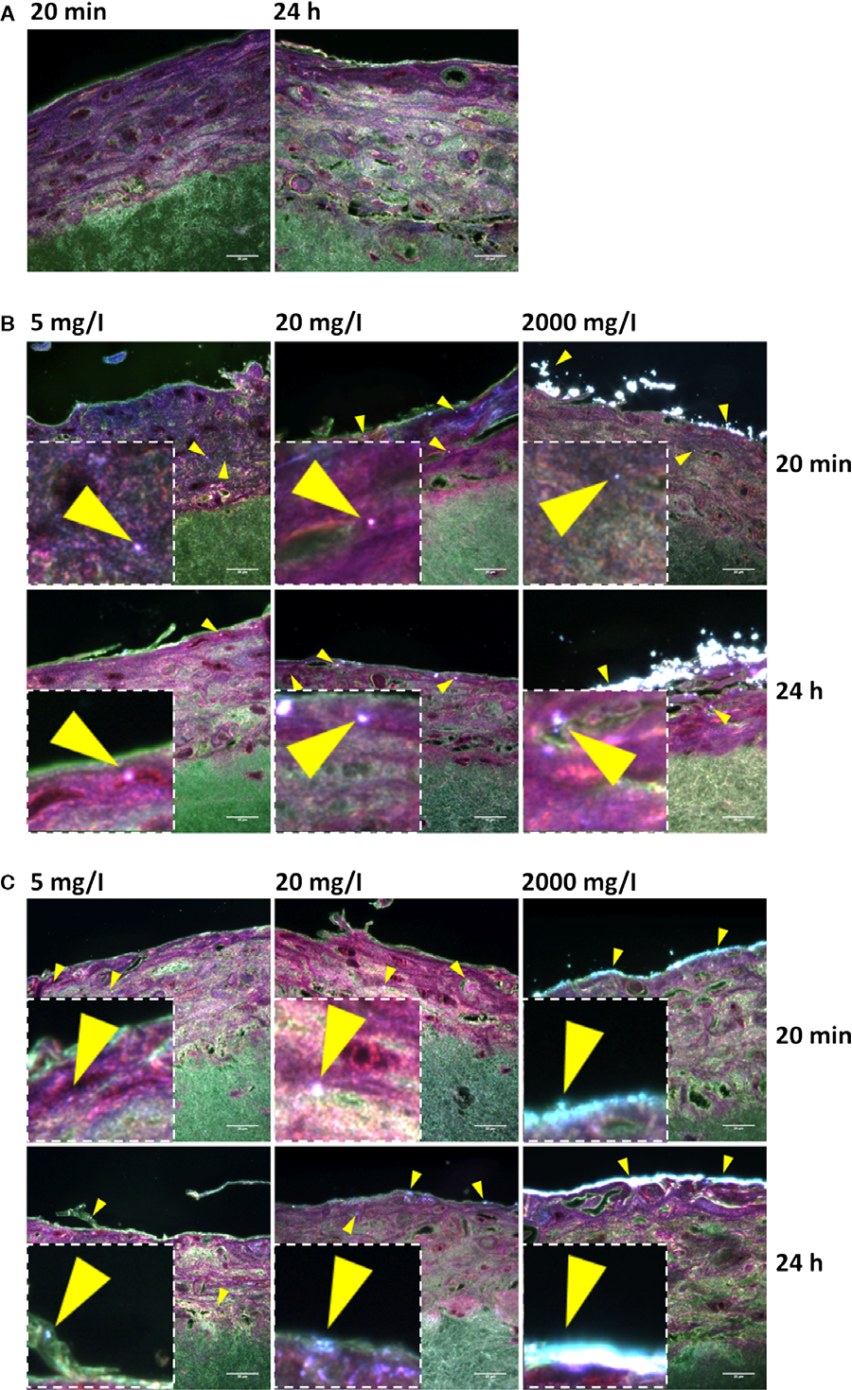
Representative URI (CytoViva) images of unexposed (A) and nano‐TiO_2_ exposed (B, C) 3D organotypic tissues for 20 min and 24 h. The reconstructed normal human oral buccal tissues were harvested, fixed in formalin, embedded in paraffin, sectioned and haematoxylin–eosin stained before URI microscopy. Nanoparticle agglomerates were identified as white/blue bright spots as indicated by arrow heads. Control (A), spherical nano‐TiO_2_ (B), spindle nano‐TiO_2_ (C). Scale‐bar: 20 μm. Inserts show zoom out of the regions of interest, where white/blue bright spots were mainly present.

**Figure 4:**
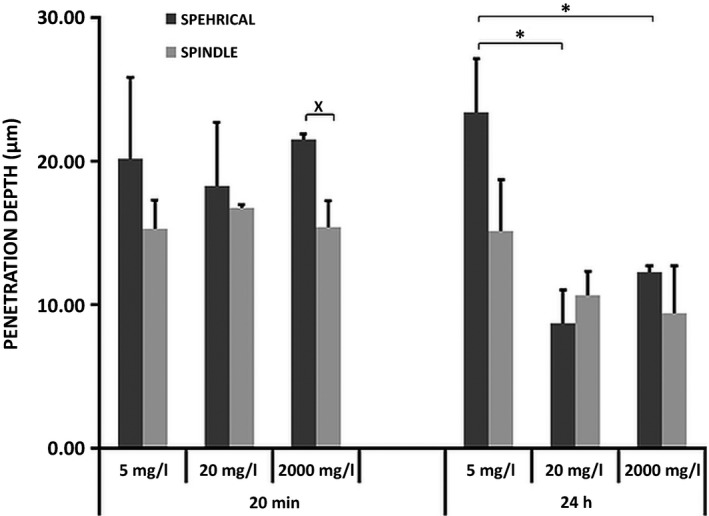
Location **(**penetration depth) of spherical‐ and spindle‐shaped nano‐TiO_2_ into the epithelium of RNHBM after 20 min and 24 h of exposure. Statistical significant difference in the depth of penetration between the different concentrations of spherical nano‐TiO_2_ is marked by stars (_*_). Statistical significant difference in the depth of penetration between the two shapes of nano‐TiO_2_ is marked by cross (_**˟**_).

All reconstituted tissues displayed a well‐differentiated stratified squamous epithelium with a distinct basal, spinous and superficial non‐keratinized cell layer, on top of a connective tissue equivalent, similar to native human buccal mucosa (Fig. 2A). Nano‐TiO_2_ agglomerates were visualized as bright white/bluish objects by URI due to their high refractive index (Fig. 3B,C) and were observed both over and within the epithelium. Larger agglomerates were seen over the epithelial layer at higher NP concentrations. At the highest concentration, an almost continuous bright belt of NP agglomerates covered the epithelium (Fig. 3B,C). Both types of nano‐TiO_2_ were identified by URI within RNHBM epithelium at early (20 min) as well as late (24 h) time points. Generally, spherical NPs were found deeper in the epithelium compared to spindle‐shaped (*P* = 0.034) (Fig. 4), although the difference was statistically significant only for the highest concentration at the early time of exposure (*P* = 0.031).

### Penetration of nano‐TiO_2_ was dose‐dependent only at later (24 h) but not at early (20 min) time point

The location of NPs within epithelium was similar for both types and all concentrations at the early time point (20 min) (*P* > 0.05) (Fig. 4). After 24 h, concentration‐dependent differences were observed. Thus, spherical nano‐TiO_2_ showed the deepest location at 5 mg/l concentration, and this was significantly deeper than at 20 and 2000 mg/l (*P* = 0.006 and *P* = 0.023, respectively). The same trend was observed for spindle‐shaped NPs, although the difference was not statistically significant.

### Penetration of nano‐TiO_2_ was time dependent at high concentrations (20 and 2000 mg/l) only

**Figure 5:**
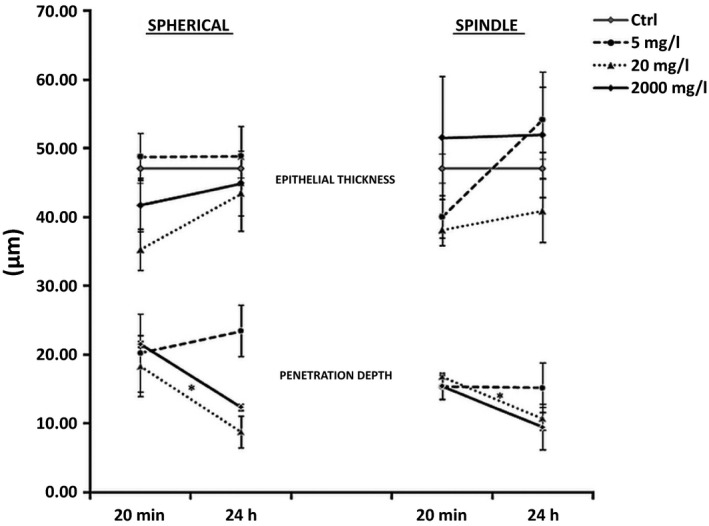
Time‐dependent variations of penetration depth of nano‐TiO_2_ particles and epithelial thickness of RNHBM exposed to nano‐TiO_2_. Statistical significant difference in the depth of penetration with time is marked by stars (_*_).

The level of epithelium penetration did not change with exposure time at the lowest concentration, regardless of NP shape. A significant decrease in the penetration depth was observed at 24 h at higher concentrations (Fig. 5) for both types of particles (*P* = 0.04). The difference was statistically significant for spherical NPs at 2000 mg/l (*P* = 0.001) and spindle‐shaped at 20 mg/l (*P* = 0.022).

### Tissues exposed to nano‐TiO_2_ showed increased desquamation but preserved epithelial thickness

Tissue architecture and microanatomy was not altered by exposure to nano‐TiO_2_ (Figs 2 and 3). At higher concentrations, increased desquamation of the superficial epithelial layers was observed, especially for tissues exposed to spherical nano‐TiO_2_ (Figs 2 and 3). Epithelial thickness of RNHBM tissues was either constant or increased after 24 h of exposure to both types of nano‐TiO_2_, although the change was not statistically significant (Fig. 5).

## Discussion

To our knowledge, this is the first study that assessed the penetration of nano‐TiO_2_ into a human buccal mucosa 3D organotypic model. Employment of a 3D model has the advantage of reconstructing in a standardized and reproducible manner the architecture of original tissue, allowing thus cell‐to‐cell and cell‐to‐matrix interactions and bringing the experimental context as close as possible to the *in vivo* settings. Nevertheless, although superior to monolayer experimental systems, the 3D organotypic model used in this study lacks vascular and immunocompetent compartments that play an important role in epithelial barrier functions 16. Moreover, *in vivo*, the epithelium of oral cavity is moistened with saliva, known to have protective properties and contain mucus that has an impact on the mobility of NPs. Thus, the 3D organotypic model used here might have been more susceptible to penetration by nano‐TiO_2_ and might have undergone more severe modifications after exposure than native tissues. However, recent studies have shown that smaller NPs, *that is* around 20 nm, in contrast to 200 nm, are able to penetrate through the layer of mucins precipitated on the surface of oral epithelium *in vivo* 17. This indicates that for smaller NPs, the effects seen *in vitro* on the OTs may actually be representative for the *in vivo* effects.

The DLS measurements showed relatively large agglomerate sizes, especially for spherical nano‐TiO_2_, which increased with concentration. The NP ζ‐potential may explain the agglomeration degree, as spherical particles had significantly lower ζ‐potential values compared to the spindle‐shaped (Table 1). However, smaller agglomerates of nano‐TiO_2_ might be present in suspensions although not detected via DLS 18, which may explain why we could detect NPs as deep as one‐third within the epithelium at all concentrations for both NP types.

Although smaller agglomerates were associated with spindle‐shaped particles_,_ they were located more superficially in the epithelium compared to the spherical. Similar findings were obtained by other research groups who reported that spindle‐shaped particles were taken up less by cells as compared to the spherical of the same crystal structure 6, 19. The lower uptake of spindle‐shaped particles has been suggested to be associated with their high aspect ratio, as it takes longer time for a cell membrane to wrap around the elongated particles 20. It has been well established that the shape plays an important role in the nano‐TiO_2_ agglomeration in biological media 12, as well as on the tissue and cellular response 11, 21.

The URI analysis revealed that both nano‐TiO_2_ types, regardless of concentration, were located approximately at the same level within epithelium after 20 min of exposure (Fig. 4). After 24 h, tissues exposed to 5 mg/l displayed NPs located deeper compared to those of 20 and 2000 mg/l. It has been shown that the concentration has significant influence on the rate of agglomeration, which increases over time in suspensions with high concentrations 12. As known, larger agglomerates have less potential to penetrate the epithelial barrier 17. Nevertheless, as indicated by our results, another important factor contributing to the more superficial location observed at higher concentrations with time might be the desquamative response of the tissues as RNHBM is not passive membranes, but biologically active systems. Unfortunately, the degree of desquamation could not be quantified, but its presence was clearly associated with exposure to nano‐TiO_2,_ especially at higher concentrations. Desquamation is one of the most efficient modalities through which stratified squamous epithelia of the body, including buccal mucosa, eliminate potentially harmful agents 22. Similar to native buccal epithelium, RNHBM has a stratified epithelium that maintains its homoeostasis by the balance between basal cell proliferation and cell loss at the surface (desquamation) 13. The fact that epithelial thickness remained the same over time after exposure despite increased cell loss (desquamation) indicates an increase in basal cell proliferation of exposed RNHBM to compensate this cell loss. These findings suggest that release of inflammatory cytokines and chemokines via MAP Kinase and NF‐κB signalling cascades might be a possible outcome of the cellular exposure to nano‐TiO_2_, leading to the increased epithelial tissue proliferation observed in this work 23, 24. Nonetheless, these findings do not exclude that proliferation might as well have been a compensatory epithelial tissue reaction due to a direct effect on the genetic components of the cells, as previous reports showed that nano‐TiO_2_ is able to induce DNA strand breaks and genetic instability 24. This increased reactive proliferation is part of the physiological barrier function, but due to wide use of nano‐TiO_2_ in a variety of products, one might expect that repeated exposures may either exhaust the proliferative capacity of epithelium, finally resulting in epithelial atrophy, or will lead to cell transformation and malignancy due to accumulation of replication errors as a consequence of a prolonged increased proliferation 25.

The permeability and the barrier function of stratified epithelial tissues have been long attributed mainly to superficial layers of the tissue 26. Thus, once a foreign object goes through the top layer of epithelium (crosses the intercellular lipids secreted by the membrane‐coating granules on the superficial layers), it is highly likely that this object would reach the underlying connective tissues 8. In the present study, both types of nano‐TiO_2_ particles crossed the superficial epithelial layers regardless of exposure time and concentration, indicating that in general, particles are able to penetrate the oral epithelial barrier in a relatively short time if they are at the right size. Nevertheless, nano‐TiO_2_ particles located in the connective tissue equivalents were found quite rare at any concentration or time point, and together with the finding that the NPs were found even more superficially after 24 h, on the way to be eliminated from the epithelium, dismisses the theory of Caon et al. 8. However, the presence of particles at deeper levels at lower concentrations suggests that due to a lower degree of agglomeration, low doses may be actually more toxic, as also indicated by previous *in vitro* studies 27.

Objects penetrate epithelial barriers by one of two routes: trans‐cellular and para‐cellular 8. Uptake of nano‐TiO_2_ by buccal epithelial cells was reported to take place as soon as 10 min after exposure, their internalization via endocytosis and then trans‐cellular transport enabling them to travel fast and deeper into the tissues 28, 29. Microplicae were reported to play an important role in NPs/agglomerates internalization into epithelial tissues in a size‐dependent manner meaning that objects need to be small enough to fit in the furrows (200–400 nm) 28, 30. Although we did not investigate the route of transport within epithelium, the location of nano‐TiO_2_ below the upper third of epithelium within 20 min after exposure suggested a para‐cellular way of transport, as it would have taken much longer for the NPs to reach this far down via a trans‐cellular route alone.

## Conclusion

In summary, nano‐TiO_2_ penetrated a reconstituted human normal buccal epithelium very soon after exposure. The penetration was shape‐, dose‐ and time‐dependent, and most of the particles remained within the upper third of the epithelial tissue. Maintained epithelial thickness despite increased desquamation at higher concentrations and longer exposure time indicates that nano‐TiO_2_ particles induced a biological response in the epithelium. Although within physiological limits after single exposures, these effects might trigger changes with adverse consequences in the long term, indicating the need for further characterization of the effects of nano‐TiO_2_ exposure.

## Funding

This work was supported by the EC FP7 NANoREG (Grant Agreement NMP4‐LA‐2013‐310584), Patient and Community Based Clinical Dental Research Group, Department of Clinical Dentistry (M.C. Marthinussen, A. Bårdsen); UH‐Nett Vest grant (M.R. Cimpan), Bergen Medical Research Foundation (D.E. Costea, 20/2009); Helse Vest (D.E. Costea, 911902/2013).

## Conflict of interest statement

None to declare.
